# Research Progress on Stimulus-Responsive Polymer Nanocarriers for Cancer Treatment

**DOI:** 10.3390/pharmaceutics15071928

**Published:** 2023-07-11

**Authors:** Shicui Luo, Zhuo Lv, Qiuqiong Yang, Renjie Chang, Junzi Wu

**Affiliations:** 1 Yunnan Key Laboratory of Integrated Traditional Chinese and Western Medicine for Chronic Disease in Prevention and Treatment, Yunnan University of Chinese Medicine, Kunming 650500, China; luoshicui@ynutcm.edu.cn (S.L.);; 2 Key Laboratory of Microcosmic Syndrome Differentiation, Yunnan University of Chinese Medicine, Kunming 650500, China; 3 Center of Digestive Endoscopy, The First Affiliated Hospital of Yunnan University of Chinese Medicine, Kunming 650021, China

**Keywords:** stimulus-responsive, polymer nanomaterials, carrier, cancer

## Abstract

As drug carriers for cancer treatment, stimulus-responsive polymer nanomaterials are a major research focus. These nanocarriers respond to specific stimulus signals (e.g., pH, redox, hypoxia, enzymes, temperature, and light) to precisely control drug release, thereby improving drug uptake rates in cancer cells and reducing drug damage to normal cells. Therefore, we reviewed the research progress in the past 6 years and the mechanisms underpinning single and multiple stimulus-responsive polymer nanocarriers in tumour therapy. The advantages and disadvantages of various stimulus-responsive polymeric nanomaterials are summarised, and the future outlook is provided to provide a scientific and theoretical rationale for further research, development, and utilisation of stimulus-responsive nanocarriers.

## 1. Introduction

Cancer is caused by abnormal cell growth and proliferation, with current incidence and death rates continuing to rise. Based on the global cancer data released by the International Agency for Research on Cancer of the World Health Organization, 19.29 million new cancer cases and 9.96 million deaths were recorded globally in 2020; by 2040, it is estimated that global cancer rates will reach 28.4 million cases, an increase of 47% from 2020 figures [1]. Thus, cancer is considered one of the major diseases endangering human health globally. Chemotherapy is a standard clinical treatment and includes common drugs, such as methotrexate (MTX), doxorubicin (DOX), paclitaxel (PTX) and camptothecin (CPT). However, limitations on their use are prevalent and include poor drug solubility, multiple drug resistance, weak tumour-targeting, short blood circulation times, low bioavailability and toxic side effects to normal cells [2]. For these reasons, safe and efficient treatment methods must be identified and developed.

Owing to considerable developments in nanomedicine, nanomaterial drug delivery systems have positively impacted anti-tumour therapy. Commonly used nanocarrier materials include liposomes, polymers, metal nanoparticles (NPs), carbon nanomaterials and mesoporous silica materials [3]. Notably, polymeric materials are an important class of biomaterials that specifically interact with biological systems for therapeutic or diagnostic purposes [4]. It has been reported that adjusting molecular composition and polymer structures enhances several desired properties, such as biodegradability, biocompatibility and mechanical strength [5]. Polymer materials have been used in implant surgical instruments [6], equipment for in vitro treatments, wound dressings [7] and drug release systems [8]. Importantly, they have made good progress in these fields. In cancer settings, nanopolymer material carriers exhibit good compatibility between hydrophilic and hydrophobic drugs and biomolecular preparations. The size of nano is usually between 10 and 200 nm [9], and the internalisation degree of the nanostructures in tumour cells is 3–5 times higher than that of micro-nanostructures [10,11]. Meanwhile, the small size of nanostructures can take full advantage of the enhanced permeability and retention effect of tumour tissue to promote drug absorption [12,13]. More notably, nanostructures can load or encapsulate multiple drugs to achieve synergistic therapeutic effects of drugs. Although various stimulus-responsive polymer nanocarriers have similar size ranges, each has unique characteristics in terms of their impact on performance. For example, the smaller size helps to increase the efficiency and stability of redox reactions, improves the binding and catalytic efficiency of enzymes, helps to penetrate into low oxygen environments, enhances the release rate and biological activity of drugs [14] and provides higher temperature sensitivity and optical effects. Therefore, when selecting and designing nano, researchers need to comprehensively consider application requirements and material properties and further study and evaluate the impact of their size on performance.

To achieve targeted anti-cancer treatment, several targeted tumour treatment measures have been developed. One of the more widely studied anti-tumour measures is exploiting differences between tumour and normal tissue or external stimuli to control precise drug release. Stimulus-responsive polymeric nano-delivery systems enable precise drug release, thereby improving drug utilisation and reducing toxic effects on normal cells. Additionally, studies have reported the use of stimulus-responsive nanocarrier materials to reduce multiple drug resistance issues [15,16,17]. In recent years, biological materials (e.g., bovine serum albumin (BSA) [18], gelatin [19], zein [20,21], polycaprolactone (PCL) [22], polylactic acid (PLA) [23,24], chitosan (CS) [25], ultrahigh-molecular-weight polyethylene (UHMWPE) [26]) have been widely used in stimulus-responsive nanoresearch systems due to their good biological characteristics. This is a very promising direction; however, in terms of nanocarrier materials, this review is more oriented towards synthetic polymeric materials. Based on synthetic polymeric nanomaterials, we reviewed the chemical bonds, functional groups and/or polymer materials commonly used in stimulus-responsive polymer materials with respect to pH, redox, enzyme, hypoxia, temperature and photo-stimulus in order to unravel their mechanisms in the past 6 years studies. Additionally, we provide brief explanations of different stimulus-responsive polymer nanomaterials. Finally, we analysed the advantages and shortcomings of various types of stimulus-responsive nanomaterials in the past 6 years and created an outlook on the future development of stimulus-responsive nanocarrier systems. Through this review, we hope to make a summary of stimulus-responsive nanomaterials in the field of cancer in the past 6 years, provide some ideas and methods and point out the direction for further research in this field.

## 2. Single Stimulus-Responsive Polymer Nanocarriers

Based on function, we can classify stimulus-responsive nanocarriers into single stimulus and multiple stimulus-responsive systems. Based on action pathways, we can classify single stimulus-responsive nanocarriers into exogenous and endogenous stimulus-responsive systems.

### 2.1. Endogenous Stimulus-Responsive Nanocarriers

At different levels, endogenous polymeric nanocarriers primarily exploit differences between the tumour microenvironment and normal tissue to control targeted nanocarrier release in tumour tissue. Internal stimuli mainly consist of pH, redox, enzymes and hypoxia.

#### 2.1.1. pH Stimulus-Responsive Polymeric Nanocarriers

In the tumour microenvironment, ATP hydrolysed products and lactic acid are produced as a result of abnormal tumour cell proliferation [27] and lead to tumour tissue pH levels of 6.2–7.0. The pH of normal tissue is 7.4 and 4.5–5.0 in lysosomes [28]. For this reason, pH-responsive nanomaterials can control drug release using pH differences between normal tissue, tumour tissue and lysosomes. In recent years of research, we have found that polymer materials containing cleavable chemical bonds or ionisable groups in acidic environments have been prioritised by several researchers.

For example, the hydrazone bond is stable in blood circulation and is easily hydrolysed in internal/lysosomal pH environments [29]. Thus, hydrazone bonds are often used to couple drugs into polymeric material structures. Liao et al. [30] designed a pH-responsive NP to connect hyaluronic acid (HA) and DOX via a hydrazone bond. This NP has a good size (167~220 nm). DOX release under different pH conditions showed that these NP-containing hydrazone bonds not only prevented premature DOX release but also accelerated its release into tumour tissue. In another study, Liu et al. [31] combined triphenylphosphine (TPP) modified novel DOX derivatives (DOX-TPP) with HA via hydrazone bonds to generate HA-hydra-DOX-TPP NPs (size ~192 nm). In an acidic environment, hydrazone bonds in NPs were cleaved, the structure disrupted, and DOX effectively released from the HA-hydro-DOX-TPP adduct, which in turn enhanced DOX accumulation in the mitochondria. These polymeric NPs demonstrated good biocompatibility and low cytotoxicity to normal cells. More importantly, acid stimulus responsiveness caused drugs to be increasingly targeted and inhibitory to tumour cells/tissue, thus enhancing drug utilisation. These studies proved that hydrazone bond addition to polymer materials facilitated the preparation of acid-sensitive materials.

**Scheme 1:**
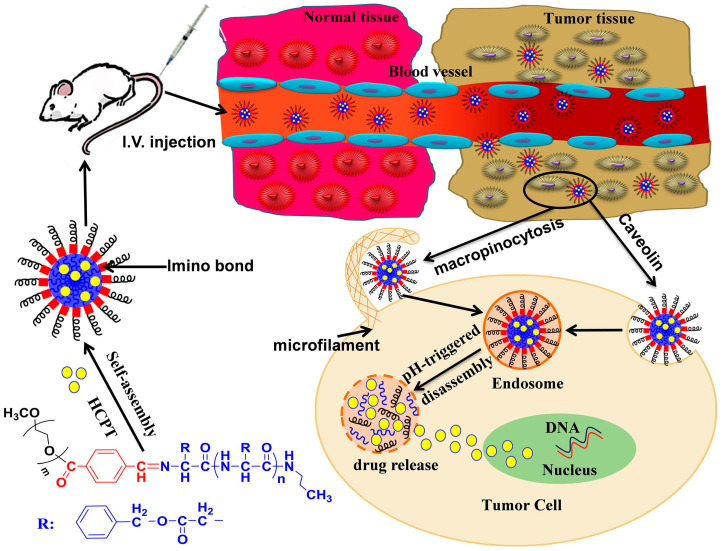
Targeted delivery strategies and polymer micelle mechanisms. Reprinted with permission from [32]. Copyright 2019, Elsevier.

The imine bond is also an acid-sensitive chemical bond; it remains stable in a neutral environment and is prone to hydrolysis under acid conditions. Based on these functions, Pu et al. [32] designed and synthesised amphiphilic polyethene glycol (PEG) imino poly aspartic acid (PIPA) polymer micelles (PIPAH). The average diameter of the PIPAH micelles was approximately 230 nm. The micelles were connected via imine groups and encapsulated hydroxy camptothecin (HCPT) (Scheme 1). HCPT-loaded micelles exhibited longer circulation times and better anti-tumour effects compared with HCPT solutions. In another study, Liao et al. [33] prepared a PEI-g-mPEG copolymer containing acid-sensitive benzoic acid-imine bonds using Schiff-base reactions of the primary amine and aldehyde groups in branched PEI and mPEG-CHO segments. The system cross-linked with hydrophobic terephthalic acid molecules in one aqueous solution (pH 7.4) step to form benzoic acid-imine cross-linked functionalised polymer nanogels (size 129 nm), which functioned as indocyanine green (ICG) delivery carriers. Under acidic conditions, the breaking of benzoic acid-imine bonds promoted ICG release. ICG-loaded nanogels improved ICG photostability in phosphate buffer and reduced ICG leakage. Hsu et al. [34] prepared pH-sensitive conjugates using acid-sensitive benzene imine bonds formed between a primary amine and aldehyde groups in PEG chitosan and 4-(dodecyl oxy) benzaldehyde DBA, which self-assembled to form CPDMs in aqueous solution (pH 7.4). At acidic pH, the imine bonds were cleaved, the drug release rate was accelerated, and the anti-tumour effect was enhanced. These studies showed that benzoic acid-imine had good acid sensitivity, with associated nanocarriers showing good stability and anti-tumour effects.

Introducing acidic or alkaline ionisable groups into polymers is one way to design pH-responsive drug delivery systems [30]. Ionisable functional groups are protonated or deprotonated at different pH values. For example, polymer carriers containing carboxylic acid, sulphonic acid and other acidic functional groups can be dissociated and deprotonated under high pH conditions [35]. This is due to electrostatic repulsion between chains, leading to water uptake, higher charge density, polymer swelling and loaded drug release. Guo et al. [36] prepared a peptide-based amphiphilic polymer containing a hydrophilic sericin main peptide chain and PBLG side chain using the ring-opening polymerisation (ROP) method. This filamentous PBLG/DOX micelle has a size of 110 nm and possesses good drug loading rate. Sericin contains many aspartic acid residues, the carboxyl groups of which are protonated under acidic conditions. Therefore, under acidic conditions, the electrostatic repulsion between sericin PBLG micelles decreased, and the micelles swelled, leading to increased DOX release rates.

Alkaline functional groups, such as pyridine derivatives, piperazine and amino salt groups, can use electricity ionisation under low pH conditions and increase charge exclusion between adjacent alkali bases [35]. Thus, polymers expand and release drugs. Ray et al. [37] designed PEG-b-poly(carbonate) block copolymers and added tertiary amine side chains to polycarbonate segments. They used the pH-specific protonation ability of PEG-b-polycarbonate block copolymers and tertiary amines to generate stable and controllable drug nanocarriers in order to induce enhanced and targeted therapeutic drug accumulation in the Pancreatic Ductal Adenocarcinoma microenvironment.

Zwitterion polymer research has also gained considerable traction in tumour therapy. In a recent study, Liu et al. [38] synthesised two amphiphilic polyurethanes (PUC and PUN) with opposite charges by introducing carboxyl and tertiary amino groups, respectively, into polyurethane. PUC-PUN nanomicelles, with inner and outer PUN and PUC layers, respectively, were prepared using electrostatic interactions between PUC and PUN micelles. Carboxyl and tertiary amine groups in micelles were rapidly protonated under acidic pH. Additionally, charge reversal occurred, cellular uptake was enhanced, and a ‘proton sponge’ effect facilitated encapsulated drug escape from lysosomes. Collectively, introducing ionisable groups into polymers is a good strategy for designing pH-responsive nanomaterials.

**Scheme 2:**
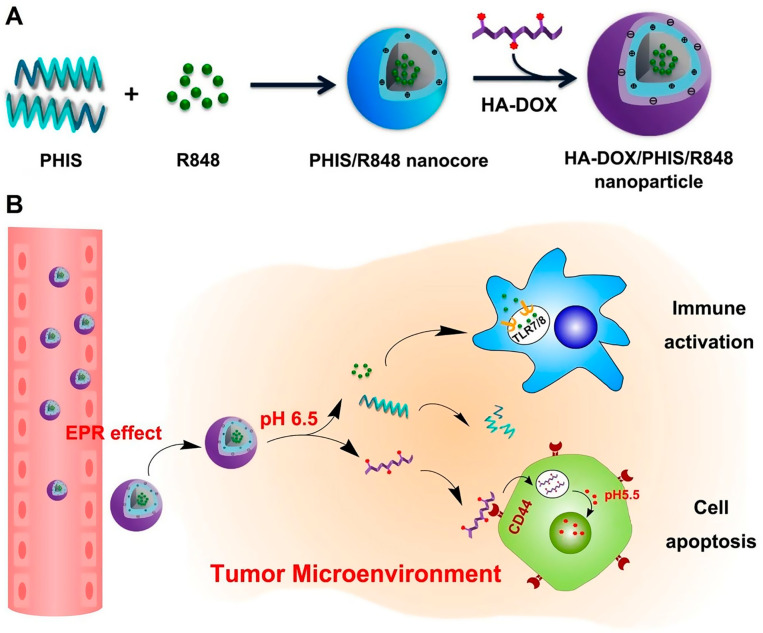
HA-DOX/PHIS/R848 NP preparation (**A**) and functional mechanisms during breast cancer treatment (**B**). Reprinted with permission from [39]. Copyright 2018, Elsevier.

To enhance pH sensitivity, researchers have developed dual pH-sensitive polymeric nanocarriers. Liu et al. [39] designed a dual pH-responsive NP combined with immunotherapy and chemotherapy-targeting therapies for breast cancer. As small molecule agonists of Toll-like receptors (TLR) 7 and 8 exhibits superior anti-tumour activity, resiquimod (R848), as a TLR7/8 agonist, has more significant induction of cytokine secretion and macrophage activation than imiquimod, a TLR7/8 agonist approved by the US Food and Drug Administration (FDA). Therefore, R848 was selected as an anti-tumour immunomodulator for this experiment. They encapsulated R848 with poly(L-histidine) (PHIS) to form PHIS/R848 nanonuclei. Then, DOX was coupled with HA via an acid-cleavable hydrazone bond to synthesise a polymer prodrug (HA-DOX), which was coated outside the PHIS/R848 nanocore to form HA-DOX/PHIS/R848 NPs. PHIS ionises around pH 6.5 and transforms from hydrophobic to hydrophilic states, triggering R848 release. Hydrazone bonds in HA-DOX break at approximately pH 5.5 (the pH of the endo/lysosome), accelerating DOX release and thus exerting cytotoxic effects. In 4T1 tumour-bearing mice, HA-DOX/PHIS/R848 NPs showed excellent tumour-targeting ability and significantly inhibited tumour growth by regulating tumour immunity and killing tumour cells (Scheme 2). Although this protocol has not yet entered the clinical phase, this dual pH NP system shows great potential in the clinical treatment of breast cancer in combination with immunotherapy and chemotherapy. In another study, Liu et al. [40] designed a dual pH-responsive bortezomib polymer conjugate (BTZ-PC) containing acetal and borate ester bonds. BTZ-PC self-assembles into micellar NPs with small particle sizes (<110 nm) and high drug-loading capacity. At acidic pHs, BTZ-PC micelle drug release rates, cell uptake, and in vitro cytotoxicity increased. These BTZ-PC micelles exhibited more potent anti-tumour effects and less in vivo hepatotoxicity compared with free BTZ. Furthermore, Liu et al. designed the desired PEG5-acetal-polycarbonate block copolymers, PC2 (PEG10k-a-poly[(TMC)_8_-co-(MTC-Cat)_24_]) and PC4 (MPEG10k-poly[(TMC)_8_-co-(MTC-Cat)_24_]). PC4 without pH-sensitive acetal linkers were used as controls. Dual pH-responsive BTZ-PC2 micelles exhibited more pronounced cytotoxicity and significantly enhanced anti-cancer effects compared with single pH-responsive BTZ-PC4 micelles in an acidic environment. Thus, this dual pH-responsive polymer nanocarrier exhibited better performances, more sensitive responses and more potent anti-cancer effects compared with a single pH response.

Polymer nanocarrier materials containing imidazole groups, carboxyl groups, ester bonds, acetals, amide bonds, borate ester bonds and maleic acid acyl groups have been widely used as pH stimulus responses (Table 1) and have shown the ability to ionise or break in the pH environment of tumours and have good pH responsiveness.

#### 2.1.2. Redox Stimulus-Responsive Polymeric Nanocarriers

Located on different reactive molecules they respond to, redox-responsive polymer materials are divided into glutathione (GSH) and reactive oxygen species (ROS). GSH types are sensitive to high GSH levels in tumour tissue, resulting in precise delivery and rapid drug release into tumour cells. Studies have shown that GSH concentrations in blood and the standard cellular matrix are 2–20 μm, whereas GSH levels in cancer cells are 2–10 mm, which is 100–500 times higher compared with the normal range [60]. Disulphide bonds may be stable in average or low-concentration GSH environments but may fracture in high-concentration environments [61]. Therefore, nanomaterials containing disulphide bonds have been used as GSH reduction-responsive nanocarriers for cancer treatment.

For instance, Sun et al. [62] designed and prepared amphiphilic polymeric nanomicelles (POEG-b-PSSDas) that contained disulphide bonds. The nanomicelles are self-assembled based on hydrophilic poly (oligomeric (ethylene glycol) methacrylate) and hydrophobic dasatinib (a carcinogenic tyrosine kinase inhibitor). Research showed that compared with PESG-b-PCCDas micelles without disulphide bonds, the difference in particle size between the two is not significant. However, DOX-loaded POEG-b-PSSDas micelles in the redox environment (10 mM GSH) exhibited faster DOX release rates, increased cytotoxicity, inhibited tumour growth more effectively, prolonged survival rates in an invasive mouse breast cancer model (4T1) and showed higher anti-tumour activity levels. Liping et al. [63] synthesised a GSH-responsive polymer (PRES) with anti-cancer activity using resveratrol (RES) and 3,30-dithiodipropionic acid as raw materials. The polymer self-assembled into NPs to encapsulate the anti-tumour drug PTX (PTX@PRES NPs). The study showed that NPs formed by PRES were stable in the systemic circulation. In the high-GSH environment in tumour cells, disulphide bonds were oxidised and reduced, with drugs released rapidly and effectively. Many researchers have performed similar studies (Table 2), which have shown that disulphide bonds can be cleaved in high-GSH environments in tumours. Critically, polymer materials containing or introducing disulphide bonds as nanocarriers have more significant anti-tumour effects and targeting properties compared with nanocarriers without disulphide bonds.

ROS is a by-product of aerobic metabolism induced by carcinogenic transformation; therefore, ROS levels in cancer cells are higher compared with normal cells. From previous reports, the main functional groups susceptible to ROS are thioacetone, thioether, selenide, diselenide, ferrocene groups and boronic acid esters.

Under ROS conditions, hydrophobic thioether-containing polymers are oxidised to hydrophilic sulfoxide or sulphone groups [68]. Thus, polymers containing thioether groups can be developed as ROS-responsive nanocarriers. Kim et al. [69] designed a thioether polymer (TEP) containing NPs (PL-TEP NPs) loaded with piperlongumine (PL). The nanomicelles had good size (187.8 ± 7.0 nm) and a high drug loading rate of (53.5 ± 2.5%). These authors investigated the effects of ROS sensitivity to TEP on drug release and anti-cancer activity. Under 100 mm hydrogen peroxide (H_2_O_2_) conditions for 8 h, approximately 95% of encapsulated PL was released from NPs, which was significantly higher compared with PBS release conditions. Similarly, the higher the degree of thioether linkage, the higher the drug release. Additionally, PL-TEP NPs had lower cytotoxicity, more efficient cellular uptake and anti-cancer activities compared with free PL.

Unlike thioethers, sulphur copper bonds are cleaved at ROS levels, disrupting nanocarrier structures and successfully releasing drugs into tumour tissue [70]. Yin et al. [70] synthesised amphiphilic block copolymer prodrugs composed of PEG and polymerised methacrylate monomers containing thioketal-linked camptothecin (CPT), which were encapsulated β-lapachone and self-assembled micelles (Lapa@NPs). A stable core–shell structure and suitable size (∼50 nm) allow Lapa@NPs to exhibit good stability under physiological conditions, possessing long circulation time and tumour aggregation properties. Lapa@NPs selectively induce a significant increase in ROS levels after tumour accumulation and internalisation into tumour cells. Enhanced ROS concentrations triggered thione linker cleavage to release the therapeutic drugs.

Ferrocene is also used to design redox-responsive polymer nanocarriers. Ferrocene groups undergo reversible changes under hydrophobic and hydrophilic conditions during redox processes, which do not alter molecular structures but only affect electron gain and loss [71]. Xu et al. [72] designed and prepared amphiphilic block copolymers plus ferrocene (PACMO-b-PAEFC). Their aim was to target cancer cells using hydrophilic poly(n-acryloyl morpholine) (PACMO) and hydrophobic poly(2-bacryloyloxyethyl ferrocene carboxylate) (PAEFC) encapsulated PTX. The size of the freshly prepared micelles ranges from 30–55 nm. Under redox conditions, electrostatic repulsion forces between ferrocene cations on side chains induced swelling or even rupture in micelle cores. Then, π–π superposition effects disappeared and permitted more rapid encapsulated PTX release from swollen micelle formulations. Thus, with increased oxidant concentrations, PTX release was increased significantly after 64 h. Copolymer micelles containing ferrocene groups improved drug efficiency or bioavailability and reduced concomitant side effects.

Moreover, for redox functional polymer materials, scientists are not satisfied with a single GSH- or ROS-type polymer nanocarrier and often choose to synthesise polymer nanomaterials that are stimulus-responsive to both GSH and ROS to enhance the targeting effect of redox stimulus response in anti-cancer treatment. The diselenide bond is sensitive to GSH and ROS levels [73,74,75] and advantageously mediates drug release in tumours. Along this direction, Kim et al. [76] designed ROS and GSH-responsive polymer nanocarriers (PD-TPP) loaded with PTX by constructing diselenide linkages and TPP. PD-TPP nanocarriers demonstrated good biocompatibility and low toxicity in normal cells. Diselenide sensitivity at high H_2_O_2_ and GSH levels was also tested. When PD-TPP (PTX) was treated with GSH or H_2_O_2_ alone, PTX cumulative release reached 80% and 65%, respectively. When GSH and H_2_O_2_ were used together, almost 100% PTX was released from PD-TPP, indicating that GSH and H_2_O_2_ increased PTX release from PD-TPP nanocarriers.

**Scheme 3:**
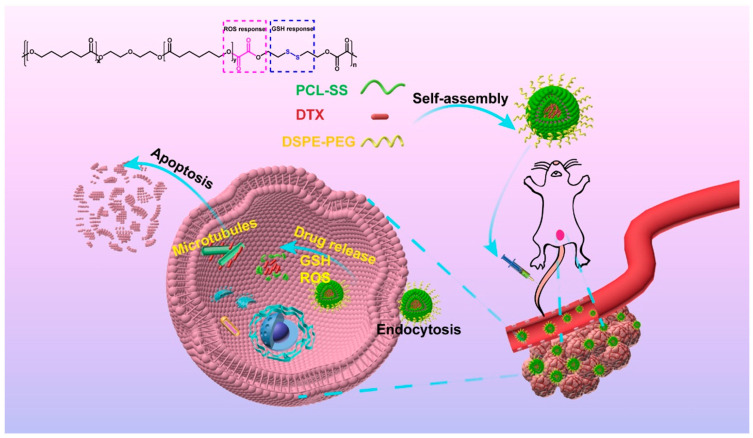
Illustration diagram of the preparation of dual-responsive PCL-SS@DTX NPs and the DTX release triggered by ROS and GSH for orthotopic prostate cancer therapy in vivo. Reprinted with permission from [77]. Copyright 2021, Elsevier.

Zhang et al. [77] developed a novel ROS-GSH dual-responsive polymer (PCL-SS) with ROS- (oxalate ester connection) and GSH-responsive structures (disulphide bond). Polymer NPs assembled with 2-hydroxyethyl disulphide, oxalyl chloride and polycaprolactone (PCL) as hydrophobic cores and DSSPE-PEG as hydrophilic shells, encapsulating DTX to form PCL-SS@DTXNPs (86 nm) (Scheme 3). Dual-responsive polymer NPs demonstrated good stability, biocompatibility and better anti-tumour effects compared with single ROS or GSH-responsive NPs. These studies fully demonstrate the advantages of ROS and GSH double-response nanocarriers and provide a research direction for redox nano-drug delivery systems.

Although previous studies reported that selenide and tellurides exhibited good ROS responses, there has been little development on ROS response carriers for selenide and tellurides, which may be attributable to biological safety issues or difficulties with carrier preparation. Nevertheless, the development potential of tellurium with ROS hypersensitivity cannot be underestimated.

#### 2.1.3. Enzyme Stimulus-Responsive Polymeric Nanocarriers

**Scheme 4:**
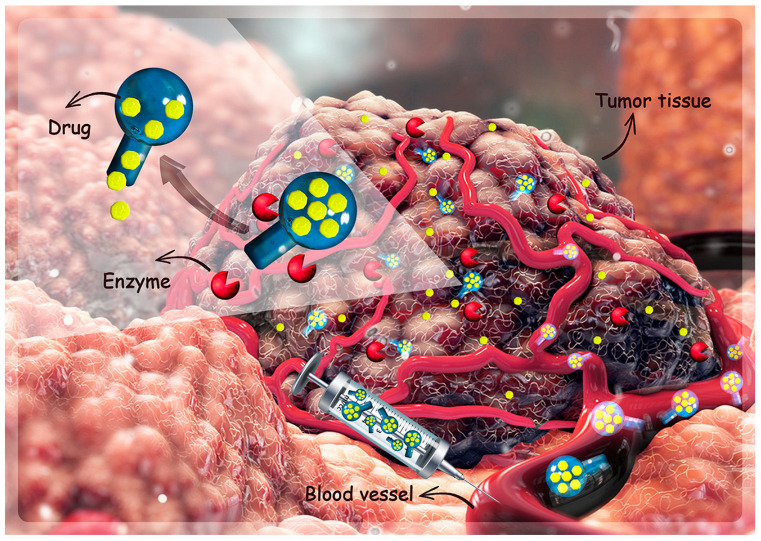
Schematic diagram of the action of the enzyme-stimulated responsive nanocarrier carrier. Reprinted with permission from [82]. Copyright 2019, Elsevier.

In recent decades, several studies have reported connections between enzymes and cancer progression. As tumour cells grow much faster than normal cells, they are more likely to invade surrounding tissue and cause abnormal enzyme expression in tumour microenvironments [78]. Enzyme production is under precise spatial and temporal control, with intrinsic structural characteristics rendering enzymes highly specific to certain substrates [79]. Therefore, enzyme properties are often exploited when designing tumour-targeting nanocarriers. The most common enzymes are matrix metalloproteinases (MMPs), phospholipase C (PLC) and cathepsin B (Cat-B) [80,81]. The mechanism of action of enzyme-stimulated responsive nanocarriers in tumour therapy is well illustrated in Scheme 4.

MMPs promote tumour proliferation, infiltration and migration [83], and it has been demonstrated that MMPs have the ability to selectively cleave peptide bonds between non-terminal amino acids [84]. Therefore, MMP traits can be used in tumour therapy. Ke et al. [85] prepared PEG-GPLGVRGDG-PDLLA by introducing an MMP-responsive peptide (GPLGVRGDG) into the biodegradable amphiphilic block copolymer poly(ethylene glycol)-b-poly(D,l-lactic acid) (PEG-PDLLA) and then self-assembled molecules into micellar for PTX loading. In the presence of MMP-2, PEG surfaces of PTX-loaded PEG-GPLGVRGDG-PDLLA (P1) micelles (average size of 62.6 ± 5.1 nm) were cleaved between G and V residues, resulting in the short peptide VRGDG functioning as a ligand to enhance cell internalisation. This study showed that P1-NPs loaded with PTX significantly enhanced 4T1 cell cytotoxicity, increased accumulation efficiency at tumour sites and considerably improved anti-tumour activity in tumour-bearing mice.

PLC overexpression enhances tumour cell proliferation and invasion [86], such that tumours may become potential targets for tumour-targeted therapy. Zhang et al. [87] reported a dendritic macromolecule with an enzyme-responsive phosphorylation structure and used it as a nanocarrier to deliver anti-cancer drugs. Phosphoramidate (PAD) dendrimers containing phosphite bonds were degraded by PLC, and DOX-loaded PAD-MPC dendrimers exhibited PLC-responsive drug release. At 20 U or 50 U PLC, drug release rates in dendritic macromolecules within 30 min were 35% and 52%, respectively. Thus, high PLC expression induced faster drug release rates. PAD-MPC dendrimers reversed drug resistance in cancer cells, prolonged blood circulation times and enhanced DOX anti-tumour effects.

Cat-B is a cysteine protease that is overexpressed in the lysosomes of different tumour cells. Studies have shown that extracellular Cat-B is involved in stromal degradation and tumour metastasis in acidic microenvironments in tumour tissue [88]. Glycylphenylalanylleucylglycine tetra-peptide (GFLG) is a suitable substrate for Cat-B; it is stable in the blood during transport and facilitates intra-lysosomal drug release [89,90,91]. Zhang et al. [89] reported a novel NP of an enzyme-responsive poly(ethylene glycosylated lysine peptide) dendrimer-gemcitabine (GEM) adduct (Dendrimer-GEM). Because GFLG acts as an enzymatic linker to GEM, binding is cleaved by lysosomal cysteine protease B at pH 6 (endosomal and lysosomal), thereby promoting more rapid GEM release. NPs exhibited enhanced anti-tumour efficacy in a 4T1 mouse breast cancer model without significant systemic toxicity but with tumour growth inhibition.

#### 2.1.4. Hypoxia Stimulus-Responsive Polymeric Nanocarriers

Hypoxia is a standard biochemical feature in solid tumour tissue [92]. In tumour microenvironments, because cancer cells are abnormally vigorous with respect to metabolism and cell growth, they consume excessive oxygen and cause angiogenic defects compared with normal cells. Cancer cells cannot provide sufficient nutrients and oxygen to the tumour ‘core’, oxygen diffusion distances are short (<200 µm) and cannot meet the oxygen demands of cancer cells that are at a distance from blood vessels [93,94,95]. Therefore, tumour tissue contains low oxygen concentration zones (PO_2_ < 10 mmHg) or hypoxia [93]. Three main strategies exist for hypoxia-targeted tumour treatments: (1) combination therapy to increase oxygen concentrations in tumours; (2) pre-bioreduction drugs conversion into active metabolites via an enzymatic reduction in hypoxia tumour environments; (3) small molecule inhibitors that modulate molecular targets involved in tumour cell survival in hypoxic tissue, such as hypoxia-inducible factor-1α (HIF-1α) [96,97]. In recent years, researchers have not limited themselves to one strategy but have combined multiple strategies to improve therapeutic feasibility and efficiency. Among the current hypoxic stimulus-responsive nanomaterials, nitroimidazole and azobenzene (Azo) derivatives have been extensively studied.

**Scheme 5:**
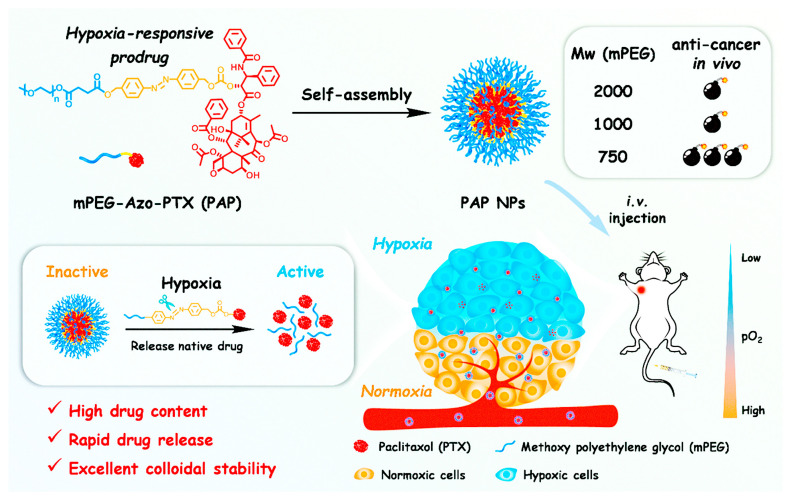
Nanoparticle (PAPNP) preparation and functional mechanisms in tumour hypoxic-specific drug release and anti-tumour activity. The number of bombs in the graph indicates the anti-tumour effect in vitro, the higher the number of bombs, the better the anti-tumour effect. Reprinted with permission from [98]. Copyright 2018, 2022 American Chemical Society.

Hao et al. [98] synthesised hypoxia-responsive PEGylated PTX prodrugs using Azo as a cleavable linker. The prodrug self-assembled into stable NPs (PAPNPs) with 26–44% drug loading rates. Azo groups in PAPNPs were rapidly reduced by overexpressed nitrate reductase and azoreductase in tumour-hypoxic microenvironments, thereby accelerating PTX release rates (Scheme 5). Thus, selective PAPNP cytotoxicity was identified under hypoxic conditions. Further, PAPNPs with shorter methoxy-PEG chains (molecular weight = 750 Da, size = 110.3 ± 1.4 nm) demonstrated enhanced anti-tumour activity and attenuated off-target toxicity.

Xie et al. [12] designed a PAMAM-AZO-PEG (PAP) polymer by combining polyamine (PAMAM) dendrimers with PEG-2000 using Azo as a linker. Loading DOX into the hydrophobic PAMAM core, HIF-1α siRNA (si-HIF) was bound to the PAMAM surface via electrostatic forces between siRNA negative ions and the peripheral cation leading amino group (PAP + DOX + si-HIF) in PAMAM. The gradual reduction in Azo groups to aniline derivatives in a hypoxic reduction environment [17]. The study confirmed that hypoxia reduced PAP. Si-HIF reversed chemoresistance by inhibiting HIF-1α expression and enhancing DOX effects. Thus, facilitating DOX and siRNA co-delivery via hypoxia-responsive size-contracting nano-repair to enhance deep DOX penetration into tumours and inhibit HIF-1α expression is an emerging strategy that interferes with hypoxia-induced cancer development. 

### 2.2. Exogenous Stimulus-Responsive Nanocarriers

Unlike endogenous nanocarriers, exogenous nanocarriers mainly function via external stimulus to release drugs into tumour tissue and mainly include light, temperature, ultrasound and magnetic approaches.

#### 2.2.1. Temperature Stimulus-Responsive Polymeric Nanocarriers

Temperature differences between tumour and normal tissue and tumour tissue sensitivity to high temperatures facilitate temperature conditioning in tumour therapy [99]. Investigations have been conducted on using temperature to control drug release during cancer treatment. The phenomenon that results when temperature induces changes in temperature-responsive material structures and solubility is called the critical dissolution temperature, which is classified into upper critical solution temperature and lower critical solution temperature (LCST) [100,101]. The shrinkage type of LCST is widely studied based on human body temperature characteristics [102]. In 1967, Scarpa et al. [103] discovered that poly(n-isopropyl acrylamide) (pNIPAM) had an LCST of 31 °C. As a result, LCST was widely investigated, being close to human body temperature (37 °C) [104,105].

In one study, Su et al. [106] designed thermosensitive nanoparticles (TNPs) composed of a copolymer with a long segment of n-isopropyl polylactic acid and assembled TNPs in an aqueous solution. Shikonin was wrapped in a degradable inner core to form shikonin-loaded TNPs (STNPs) with a volume phase transition temperature of approximately 40 °C. Micelles safely encapsulated anti-cancer drugs at approximately 37 °C and were stable in circulation. However, when micelles reached tumour sites, which were 3–5 °C higher than average tissue temperatures, the shell changed from a hydrophilic to a hydrophobic state, and shikonin was released into the cytoplasm of breast cancer cells. Moreover, STNPs (40 °C) demonstrated selective cytotoxicity toward breast cancer cells, shikonin release rates increased, and intracellular uptake increased when the temperature rose four-fold.

Luckanagul et al. [107] prepared chitosan-grafted pNIPAM (CS-g-pN) nanogels to deliver an anti-cancer drug (curcumin) using chitosan-grafted poly(n-isopropyl acrylamide) (pNIPAM). CS-g-pN nanogel formulations exhibited reasonable encapsulation rates and biocompatibility qualities and were not toxic to normal cells. In another study, Ghasemi et al. [108] prepared thermally responsive PNIPAAm-b-poly(dodecyl acrylate) (PLA) NPs. The hydrophobic internal region was loaded with DTX at a high loading rate. In vitro drug release spectra from polymer micelles indicated that DTX delivery was thermosensitive and exhibited sustained drug release rates.

Although temperature-responsive polymer nanomaterials have been extensively studied across stimulus-responsive polymer nanomaterials [109,110], current research indicates that researchers are more likely to combine temperature-sensitive materials with other materials to form dual or multiple stimulus-responsive combination therapies. This may be due to insufficient temperature differences between normal and tumour tissue to allow nanocarriers to aggregate and function specifically at tumour sites. Combined therapies can increase temperature differences within the tissue, making them more sensitive and inducing better anti-tumour effects.

#### 2.2.2. Light Stimulus-Responsive Polymeric Nanocarriers

Compared with other stimulus responsiveness traits, photo-responsive polymer nanomicelles do not require environmental changes. Both wavelength and light intensity characteristics can be precisely adjusted, while time, direction and irradiated light areas are easily controlled, with potential applications in drug-controlled release [111,112,113]. Based on light wavelength classification, photo-responsive nanomaterials are usually divided into ultraviolet (UV), visible and near-infrared (NIR) light-responsive materials [114].

In full-wave band light, UV light contains higher photon energy levels and can break chemical bonds to facilitate controlled drug release. Although researchers have used amide bonds as pH-responsive chemical bonds, the response mechanisms of both are not the same; thus, in this section, we focus on amide bond cleavage under UV conditions. Chen et al. [115] designed and synthesised amide-linked DOX and PEG conjugated polymer, which self-assembled in water to form polymeric micelles (Poly DOX-M). Under UV irradiation, amide bonds were disrupted, inducing rapid DOX release. Therefore, efficient chemotherapy drug release may be achieved using UV irradiation in tumours. Chen et al. reported that poly-DOX m fluorescence intensity increased after 3 min under UV irradiation. This indicated that UV light effectively caused structural changes in NPs, which was related to poly-DOX m photosensitivity. Additionally, micelle diameters after UV irradiation was approximately 10 nm, which may have enhanced tumour penetration. In vivo, immunohistochemistry data showed that poly-DOX m exerted no significant damage in normal organs. Thus, poly-DOX m polymeric micelles demonstrated good photosensitive response properties, cellular uptake, biosafety and anti-cancer effects.

Quinone derivatives are relatively common photosensitive materials. Kim et al. [116] established photosensitive polymer diazonaphthoquinone (DNQ) conjugated micellar NPs, which self-assembled into nanomicelles in an aqueous solution and encapsulated DTX in its hydrophobic core. Due to π–π stacking interactions between DNQ and aromatic DTX, micelles demonstrated better DTX compatibility and higher stability in bovine serum albumin solution. Under UV (365-nm) irradiation, micellar core polarity changed from a hydrophobic to a hydrophilic state and released encapsulated DTX. Exocytic studies showed enhanced DTX-loaded micellar NP cytotoxicity after UV irradiation. Enhanced stability also increased micellar NP circulation times in the bloodstream and improved its efficacy toward cancer treatment. 

O-nitrobenzyl ester groups can be cleaved to form carboxylic acids under UV light irradiation; thus, they are commonly used as photosensitive materials [117]. Consequently, Sun et al. [118] designed and synthesised a photo-responsive amphiphilic polymer (hyaluronan-o-nitrobenzyl-stearyl chain (HA-NB-SC)) where DOX was loaded into HA-NB-SC NP cores. The number average hydrodynamic diameter of nanomicelles is 139 nm. These authors monitored the light responses of NP behaviours using absorption spectroscopy. As a longer UV wavelength (365 nm) caused less cellular damage compared with shorter UV wavelengths, the longer UV wavelength was chosen for irradiation. With increased UV irradiation times, a new absorption band appeared near 330 nm and showed that HA-NB-SC NPs loaded with DOX were decomposed at 365 nm, thus releasing DOX at tumour sites.

Furthermore, due to excellent biocompatibility, biodegradability and low toxicity traits, polycarbonate (PC) can be functionalised using different functional groups for specific therapeutic applications. Therefore, the introduction of spiropyrans (SP) on PC can also be a good photosensitive material. For example, photosensitive PC diblock copolymers with SP chromophores on side chains exhibited reversible micelle transition in aqueous solution due to the photoisomerisation of SP and merocyanine forms [113].

Compared with UV light, NIR light is more tissue-penetrating and less cytotoxic [119]. Therefore, using NIR to stimulate targeted drug release is a major research topic. In addition to its role as a trigger of controlled drug release, NIR light can be used to activate photothermal conversion agents and photosensitisers to generate heat and ROS, thereby facilitating tumour photothermal therapy (PTT) and photodynamic therapy [120]. This combinatorial approach is gradually gaining increased research traction due to its promising potential in treating localised tumours. Photo-stimulus-responsive nanomaterials have also been widely developed, but light penetration into tissue is limited due to wavelength limitations. Therefore, in future research, it is expected that incident light penetration into tissue may be enhanced to improve therapeutic effects.

### 2.3. Other Stimulus-Responsive Polymeric Nanocarriers

Apart from the aforementioned stimuli, sialic acid (SA) can be overexpressed on tumour cell surfaces. Previous studies reported that SA was related to tumour progression, metastasis, apoptosis and drug resistance [121]. Therefore, SA-tumour-specific nanocarriers can be prepared to facilitate tumour-targeted therapy. PBA is an organic boric acid that functions as a molecular receptor capable of binding to compounds containing cis-glycol groups. With a diol structure, SA is recognised by PBA. Polymeric nanocarriers containing PBA materials can bind to different SA epitopes that are overexpressed on tumour cell surfaces, thus acting as tumour-targeting ligands [122,123,124,125]. In terms of SA sensitivity, Luo et al. [125] summarised SA and PBA stimulus–response mechanisms for targeted tumour treatments and reviewed the literature on PBA-based nanocarrier materials, thereby providing major insights for the development and application of more SA stimulus-responsive nanocarriers in the future.

## 3. Multiple Stimulation-Responsive Polymeric Nanocarriers

In addition to single stimulus-responsive polymer nanocarriers, multiple nanocarrier strategies have attracted considerable attention in recent years due to improved drug release rates at specific sites. This multiple-response stimulus approach can be a combination of endogenous or exogenous stimulus-responsive materials or a combination of endogenous and exogenous sensitive materials (Table 3).

For example, the pH and redox dual stimulus–response system is a well-studied dual stimulus–response system. Cai et al. [146] designed a pH/redox reaction copolymer that self-assembled into NPs (HA-hz-ss-SA). The authors’ self-assembled hydrazone and disulphide bonds linking HA and stearic acid into NPs encapsulated with cypate and AT13387. Cypate has the ability to readily generate thermal and ROS and the ability to achieve near-infrared fluorescence imaging. Tumour cells develop heat resistance after exposure to high temperatures in PPT, which later leads to HPS90 overexpressing, and AT13387 is a novel inhibitor of HPS90, which can well improve anti-cancer efficacy. HA-hz-ss-SA, containing hydrazone and disulphide bonds, was sensitive to increased GSH levels in lysosomes and tumour cytoplasm at low pH (~5.0), thereby enabling adequate AT13387 and cypate release into the tumour cell cytoplasm.

Zhou et al. [147] synthesised a novel temperature-responsive side chain photosensitive block copolymer poly[(n-isopropyl acrylamide-co-n, n-dimethyl acrylamide) block-acryloyl-4-azo-benzoate] (P(NIPAM-co-DMAA)-b-PAzoHPA) using atom transfer radical polymerisation. The encapsulation efficiency of the paclitaxel (PTX)-loaded copolymer micelles is 83.7% and has been experimentally verified. Under UV irradiation at 40 °C, PTX release was approximately 79.6%, which was higher compared with PTX release at a single temperature or under UV light conditions. This phenomenon arose due to the synergistic effects of temperature and UV, which promoted PTX release. These results suggested that dual-stimulus-responsive polymeric nanocarriers can achieve drug targeting and controlled release into tumour cells by simultaneous heating and UV irradiation, thereby improving anti-cancer effects and providing insights into anti-cancer therapy.

Dual stimulus-responsive nanomaterials with temperature and ROS sensitivity characteristics have also been developed for specific drug delivery. Tang et al. [148] combined a temperature-responsive polymer (PNIPAm) with poly(propylene sulphide) (PPS) blocks to form temperature and ROS-responsive PPS-PNIPAm diblock copolymers. PPS contains thioethers in each of its repeating units that can be used as ROS-sensitive materials. They measured DOX release under a single temperature and H_2_O_2_ changes and both conditions. At 37 °C and 0.1% H_2_O_2_, DOX release rates were faster compared with single conditions. This dual response accelerated release was explained by the fact that at 37 °C, PNIPAm coronal contraction made it easier for H_2_O_2_ to enter PPS cores, thereby enhancing oxidative reactivity and leading to synergistic-release of PPS-PNIPAm micelles in response to both stimuli. This strategy of polymerising endogenous and exogenous stimulus-responsive materials into novel polymeric nanocarriers has provided new insights into polymer design.

With considerable research on dual-responsive polymer nanocarriers, different stimulus-responsive polymer carriers have been explored. Du et al. [149] prepared FA-CSPN by complexing folate chitosan conjugates (FA-CS) poly(n-isopropyl acrylamide) (NH_2_-PNIPAM) via ester bonds. Their study showed that amino protonation reactions of CS under acidic conditions led to the expansion and dissolution of CS; when esterases rapidly hydrolysed ester bonds, the temperature rises destroyed poly(n-isopropyl acrylamide) structures. These micelles were less toxic to normal histiocytes, delayed tumour growth, inhibited cancer cell proliferation and enhanced cellular micelle uptake. Additionally, gambogic acid (GA) release rates from micelles were significantly enhanced under acidic, esterase and high-temperature conditions compared with single-stimulus conditions. These data illustrated that multiple stimulus-responsive polymeric nanocarriers significantly increased drug release rates and were excellent cancer therapy strategies.

**Scheme 6:**
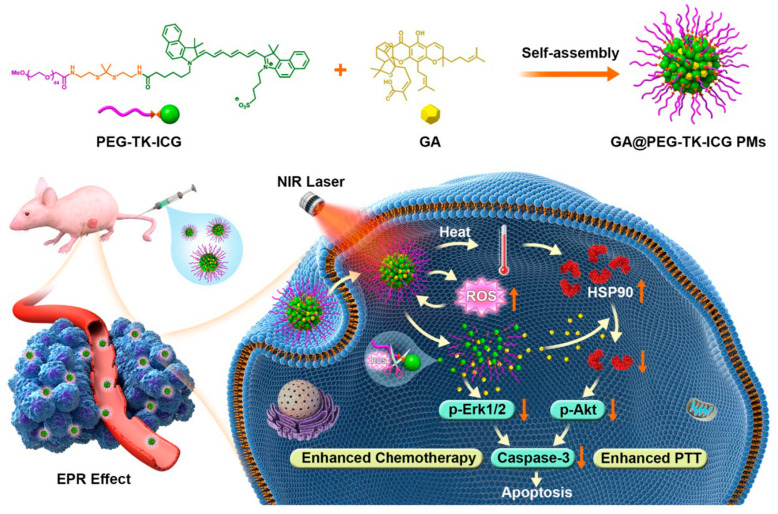
Preparation process of polymeric micelles (GA@PEG-TK-ICG PM) and some of the processes that occur in vivo in response to stimulation. Reprinted with permission from [150]. Copyright 2021, Elsevier.

Apart from the aforementioned simultaneous dual or multiple responses, several multiple dual-response nanocarrier systems exist. For example, Yang et al. [150] designed an amphiphilic polymer poly(ethylene glycol)-thione-(ICG) (PEG-TK-ICG) to prepare bioactive GA-loaded polymeric micelles (GA@PEG-TK-ICG PMs) coupled with ICG. These micelles converted absorbed light energy into PTT and ROS at 808 nm laser irradiation. Further, high ROS levels cleaved TK bonds in polymers, causing micelle decomposition and thereby facilitating rapid and controllable GA release (Scheme 6). Such micelles have higher photothermal conversion efficiency and more drug release and may provide a viable option for the effective treatment of invasive breast cancer. Irrespective of whether these different stimuli-controlled micelle drug release separately or simultaneously, the characteristics between different responsive substances were fully utilised, enabling a more precise regulation of drug-controlled release and obtaining multiple drug resistance properties for better therapeutic effects. Based on these advantages, multi-responsive polymeric nanomaterial research has attracted much attention.

## 4. Summary and Perspectives

In recent years, stimulus-responsive nanopolymer materials have received tremendous attention in the research on anti-cancer therapy. Compared with conventional therapeutic approaches, polymeric nanocarriers allow encapsulated drug release by endogenous or exogenous stimulation, resulting in altered carrier structures. This increases drug release rates, ensures no premature drug leakage occurs and achieves targeted drug therapy. Additionally, multidrug resistance has been noticeably improved. Various types of stimulus-responsive polymeric nanocarriers have achieved remarkable research progress in practical applications. pH, redox, enzyme, hypoxia, temperature and photostimulus-responsive polymeric nanocarriers have been widely studied for drug delivery, therapy and tissue engineering. Their advances in various fields offer new possibilities for realising personalised medicine and improving treatment outcomes.

In stimulus-responsive polymer nanosystems, different stimulus types have their own advantages and disadvantages. For example, pH stimulus-responsive polymer nanocarriers respond quickly and have a wide range of pH regulation. However, the values of pH in different tumour environments vary, which leads to the different effects of pH nano-stimulus response vectors in different tumours. Of course, this individual difference exists not only in pH stimulus-responsive nanocarriers, but also in other stimulus-responsive nanocarriers. Redox-stimulated responsive polymeric nanocarriers are rapidly responsive and can be used for applications such as antioxidant, tumour therapy and tissue engineering. Although the chemical bonding mechanism by which they can respond is clear, the complex preparation of stimulus-responsive nanocarrier materials and their incomplete degradation in vivo is also a challenge for this system. Enzyme-stimulated responsive polymeric nano is a good strategy for tumour treatment because it can target tumours with specific enzymes and are highly selective and sensitive. However, due to the instability of the enzyme, the drug may be released prematurely. Hypoxia-stimulus-responsive polymeric nanocarriers to treat cancer is a therapeutic strategy with great development potential. At present, the mechanism and effect of emerging strategies that combine hypoxia-stimulus–response and signalling pathways in the human body are still unknown. We look forward to further validation to provide a more feasible solution for clinical treatment. In exogenous stimulus-responsive systems, temperature-stimulus-responsive polymer nanocarriers have a wide range of temperature responses but are suitable for combining with other stimuli or therapies to achieve better therapeutic effects. Light-stimulus-responsive polymer nanocarriers have good controllability as external stimuli, but as mentioned above, UV light photons have high energy but weak penetration, and NIR light has strong penetration, but its efficiency is low, and prolonged exposure to light is required to achieve the target therapeutic effect. In recent years, studies on pH, redox and enzymes in single-stimulating polymeric nanocarrier materials have been more frequent due to the ease of preparation and transparent mechanism of action of these stimulus-responsive materials. Combined light and temperature-sensitive studies are now common among multiple stimulus–response studies. It is possible that this multiple stimulus-response can make the stimulus-response of the drug more sensitive and increase the drug release rate. Although multiple stimulus-responsive nanocarrier systems show better drug release rates and anti-tumour effects than single stimulus-responsive systems, researchers have mostly considered performance, preparation process, biocompatibility and stability to select an ideal polymeric nanocarrier. We hope that this review will provide a summary of the stimulus-responsive nanomaterials in the field of cancer treatment in the past 6 years and provide some ideas and methods for the next research in this field.

Despite promising developments, these therapies face several challenges. The deficient aspects of the various stimulus-responsive nanopolymer carriers mentioned above are issues that need to be addressed in future nanocarrier research. In addition to the aforementioned stimulating nano, other stimuli, such as electrical and magnetic targeting, have come to the fore. There are also many chemical and cytokine transitions expressed in the tumour microenvironment; it is expected that potential and promising stimulus applied to polymeric nano that targets tumours will be discovered in the near future and applied to clinical practice. In addition, during the research process, we found that currently, stimulus-responsive nanomaterials are not restricted to polymer materials. For example, in light-responsive nanomaterials, researchers often combine gold nanomaterials, which shows that in the future, novel nanomaterials may be developed as carriers in the field of nanotechnology for cancer.

## Figures and Tables

**Table 1:** pH stimulus-responsive polymeric nanocarriers in cancer therapy.

Stimulus-Responsive	Chemical Bonds or Group	Nanocarriers	Drugs	Refs.
Single pH	Hydrazone bond	PTX-loaded poly(ethyleneglycol)-phenylhydrazone-dilaurate (PEG-BHyd-dC12) micelles (PEG-Bhyd-dC12/PTX).	PTX	[41]
		TOS-DOX prodrugs (TOS-H-DOX)	DOX	[42]
		Pluronic P123-double (d)–hydrazone bond (hyd)–docetaxel (DTX) conjugates (P123-d–hyd–DTX)	DTX	[43]
		PEG-DiHyd-PLA	PTX	[44]
	Boronate ester bond	Alginate-dopamine–bortezomib (AlgPD-BTZ hydrogel)	BTZ	[45]
		mPEG-BCPGluCPT	DOX	[46]
	Ester bond	APBA and OEAM were copolymerised to obtain nanogel PEGOE-OMAP	DOX	[47]
		HDO-NPs	Dasatinib (DAS) and Olaparib (OLA)	[48]
	Acetal	Ketal containing HA nanogels	DOX	[49]
		(mPEG-b-MTC-EGVE-graft-PTX)	PTX	[50]
	Imidazole groups	Poly(ethyleneglycol)_3400_-aconityllinkage-poly(L-glutamicacid)_15_-poly(L-histidine)_10_-poly(L-leucine)_10_	DOX	[51]
		Poly N-(2-hydroxypropyl) methacry-lamid–b-poly histidine/poly leucine (pHPMA-pHis/pLeu)	PTX	[52]
	Amino acid-modified PAMAM dendritic nanocarriers	Amino acid-modified PAMAM dendritic nanocarriers	DOX	[53]
	Amide bond	Nanomicelle (QT-CA-CS) based on chitosan, quercetin and citraconic	DOX	[54]
	Maleic acid group	Anisamide-conjugated *N*-octyl-*N*,*O*-maleoyl-*O*-phosphoryl chitosan (a-OMPC)	PTX	[55]
		Polymer dimethylmaleic acid-chitosan-UA (DA-CS-UA)	DOX	[56]
Dual pH	Hydrazone and PDEA	mPEG-g-PDPP-g-hyd-DOX	DOX	[57]
	Hydrazone and Ester bonds	PEG-benzaldehyde-hydrazone-cholesteryl hemisuccinate (PEGB-Hz-CHEMS)	Gemcitabine	[58]
	β-carboxylic amide and Schiff	DCCA/DOX-NPs	DOX	[59]

**Table 2:** GSH stimulus-responsive polymeric nanocarriers in cancer therapy.

Stimulus-Responsive	Chemical Bond	Nanocarriers	Drugs	Refs.
GSH	Disulphide bonds	A cystine-bridged peptide (CBP) was designed to co-assembly with a hydrophobic anti-tumour drug curcumin (CCM) (CCM-CBP)	Curcumin (CCM)	[64]
		solanesyl poly(ethylene glycol) dithiodipropionate (SPDP)	DOX	[65]
		HA conjugated with D-α-Tocopherol Succinate (TOS) using a disulphide bond as the linker (HA-SS-TOS, HSST)	PTX	[66]
		mPEG-PLG(DNs)	DOX	[67]
		A mixed micelle system (THSP) was prepared by combining reduction-sensitive HA-poly(lactide) (HA-ss-PLA) conjugates and D-α-tocopheryl polyethylene glycol 1000 succinate (TPGS)	PTX	[15]

**Table 3:** Multiple stimulus-responsive polymeric nanocarriers in cancer therapy.

Stimulus-Responsive	Chemical Bonds or Group	Nanocarriers	Drugs	Refs.
pH and redox	Disulphide bond and carboxylic acid groups	Synthesis of PEG and polycarbonate diblock copolymers containing disulphide bonds and carboxylic acid groups (PEG-SS-COOH)	DOX	[126]
	Carboxylic acid groups and thioethers	Thioether-containing carboxylic acid- and phenylurea-functionalised PEG-block-polycarbonates were synthesised to form mixed micelles	DOX	[127]
	Amine group, carboxyl groups and disulphide bonds	The amphiphilic PEGylated poly(a-lipoic acid) co-polymer (mPEG-PaLA)	PTX and DOX	[128]
	Tertiary amine groups and disulphide bonds	Fabricated a series of mixed micelles with different mass ratios using two amphiphilic copolymers P(DMAEMA-co-MaPCL) and PCL-SS-PMPC	DOX	[129]
pH and temperature	(Diisopropyla–mino) ethyl groups and poly(N-isopropylacrylamide	Composed of a temperature-responsive corona segment with poly(N-isopropylacrylamide-co-dimethylacrylamide) and a pH-responsive core segment with poly[2-(diisopropylamino)ethyl methacrylate] (PDPA)	DOX	[130]
	Carboxyl group and poly(N-isopropylacrylamide)	Poly(N-isopropylacrylamide) (PNIPAm-NH2) with L-Aspartic acid-N-carboxyanhydride (L-Asp-NCA) to synthesise an amphiphilic PNIPAm-b-Pasp co-polymer	Methotrexate (MTX)	[131]
	Amino group and poly(N-isopropylacrylamide)	L-peptide functionalised dual-responsive nanoparticles (L-CS-g-PNIPAM-PTX)	PTX	[132]
	Amine groups and poly(N-isopropylacrylamide)	A non-toxic pH/thermo-responsive hydrogel P(NIPAAm-co-DMAEMA)	Curcumin and DOX	[133]
pH and photo	Schiff-base and o-nitrobenzyl ester group	Combining the ROP of the newly designed carbonate monomer, PEGylation, azide-alkyne Huisgen cycloaddition and Schiff-base reaction, two amphiphilic diblock polycarbonates covalently conjugated on the side chains were prepared	DOX	[134]
	Thioketal and Ce6	Thioketal cross-linked polyphosphoester-based NPs with a tumour acidity (pHe)-sensitive transactivator of transcription (TAT) peptide (DA-masked TAT-decorating reactive oxygen species (ROS)-sensitive Ce6/DOX-loaded hyperbranched NPs (DTRCD))	DOX	[135]
pH and enzymes	Poly(β-amino ester) and hyaluronidase	HA-coated self-assembly PEG-poly(β-amino ester) (PEG-PBAE) micelles	Thioridazine	[136]
	hydrazone bond and hyaluronidase	HA-Ce6 (DOX) NPs combined HA and chlorin e6 (Ce6) using adipic dihydrazide (ADH) as a linker	DOX	[137]
Redox and temperature	Disulphide linkages and poly(NIPAAm)	P(NIPAAm-co-PMA) nanocapsules	DOX	[138]
	Disulphide linkages and PVCL chains	DOX-encapsulated poly(vinyl caprolactam) (PVCL)break//	DOX	[139]
Redox and photo	Disulphide bond and o-nitrobenzyl ester derivative	Loading o-nitrobenzyl ester derivative caged DOX (DOC) into the inner poly(lactic-co-glycolic acid) (PLGA) core and adsorbing siRNA of P-gp protein onto the cationic polymeric shell derived from a disulphide-containing alkyl modified polyethylenimine (C16-S-S-PEI)	DOX	[140]
	Disulphide bond and Cypate	Proportionally mixing polycaprolactone-disulphide bond-biodegradable photoluminescent polymer (PCL-SS-BPLP) and biotin-polyethylene glycol-cypate (biotin-PEG-cypate)	DOX	[141]
Redox and enzymes	quinone oxidoreductase 1 (NQO1) and monomer (TMBQ)	polyurethane (PEG-PTU-PEG) block copolymer was designed and synthesised by co-polymerising diisocyanate, a reduction-sensitive monomer (TMBQ) and poly(ethylene glycol)	DOX	[142]
Enzymes and temperature	Acetylcholinesterase (AchE) and the long carbon chains of LCC	Prepared based on seven-[6-(diaminodipropylamine)-6-deoxy]–β-CD (SA- β-CD) and lauroyl choline chloride (LCC)	Celastrol (CSL)	[143]
Temperature, pH and reduction	poly(N-isopropylacrylamide), carboxylic acid groups and tertiary amine groups, disulphide bonds	tetrablock co-polymer poly(polyethylene glycol methacrylate)–poly [2-(dimethylamino) ethylmethacrylate]–poly(N-isopropylacrylamide)–poly(methylacrylic acid) (PPEGMA-PDMAEMA-PNIPAM-PMAA)	DOX	[144]
	PNIPAM, disulphide linkage, acetal-bridged	A comb-like copolymer (G3) comprising one disulphide linkage and PEG, PCL and acetal-bridged PCL-b-PNIPAM grafts	DOX	[145]

## Data Availability

Not applicable.
